# Physiological changes after deflation of Resuscitative Endovascular Balloon Occlusion of the Aorta following Automated Head-up Position Cardiopulmonary Resuscitation: a post-hoc analysis

**DOI:** 10.1016/j.resplu.2026.101370

**Published:** 2026-05-27

**Authors:** N. Segond, J. Moore, P. Pourzand, B. Salverda, M. Suresh, K. Bachista, A. Johnson, S.T. Youngquist, G. Debaty, K. Lurie

**Affiliations:** a Univ. Grenoble Alpes, Emergency Department and Mobile Intensive Care Unit, CNRS, UMR 5525, VetAgro Sup, Grenoble INP, TIMC, 38000 Grenoble, France; b Department of Emergency Medicine, University of Minnesota, Minneapolis, MN, USA; c Hennepin Healthcare Research Institute, Minneapolis, MN, USA; d Department of Medicine, CentraCare-St. Cloud Hospital and CentraCare Regional Campus St. Cloud, St. Cloud, MN, USA; e Department of Emergency Medicine, Lehigh Valley Health Network, Bethlehem, PA, USA; f Mayo Clinic School of Health Sciences, Mayo Clinic in Florida, Jacksonville, FL, USA; g Department of Emergency Medicine, University of Utah Health, Salt Lake City, UT, USA

**Keywords:** Cardiopulmonary resuscitation, Endovascular procedures, Intra-aortic balloon, Resuscitation, Resuscitative endovascular balloon occlusion of the aorta

## Abstract

**Background:**

Use of Resuscitative Endovascular Balloon Occlusion of the Aorta (REBOA) and the automated head-up position (AHUP) have increasingly both been separately reported during cardiopulmonary resuscitation (CPR). The optimal hemodynamic management of REBOA balloon deflation after the return of spontaneous circulation (ROSC) is unknown. We hypothesized that partial deflation of REBOA rather than full deflation after ROSC would result in better hemodynamic parameters.

**Methods:**

After 10 min of untreated ventricular fibrillation, AHUP-CPR was performed on 20 pigs (weighting ∼40 kg). After 36 min, a REBOA balloon was inflated. After ROSC (13 pigs) REBOA deflation was initiated in one of two ways: complete (100%) or partial (50%) deflation. The primary endpoint was mean difference in mean aortic pressure (MAP) compared one minute before and one minute after REBOA deflation. Secondary endpoints were mean difference in cerebral perfusion (CerPP) and coronary perfusion (CorPP) pressures (in mmHg). Data were compared using Mann-Whitney *U* test.

**Results:**

After ROSC the mean difference in MAP before and after deflation was 38.5 mmHg (95%CI: 17.0–60.0) with complete deflation versus 7.0 (95%CI: 1.8–12.3) with partial deflation (*p* = 0.006). The mean difference in CerPP was 36.3 (95%CI: 15.9–56.8) with complete deflation versus 7.1 (95%CI: 1.7–12.5) with partial deflation (*p* = 0.01) and the mean difference in CorPP was 38.0 (95%CI: 18.7–60.9) with complete deflation versus 6.9 (95%CI: 0.9–11.2) with partial deflation (*p* = 0.006).

**Conclusion:**

In this porcine model of prolonged AHUP-CPR, partial REBOA balloon deflation resulted in superior hemodynamics compared with complete deflation.

## Introduction

Management of out-of-hospital cardiac arrest (OHCA) remains an ongoing challenge in critical care, as survival rates remain poor despite some advances in cardiopulmonary resuscitation (CPR) techniques.^1^ Survival and favorable neurological outcomes after cardiac arrest are critically dependent on cardio-cerebral blood flow during CPR.^2^, ^3^ Studies spanning a more than a half century have consistently shown that cerebral perfusion (CerPP) and coronary perfusion (CorPP) pressures are inherently limited during conventional (C) CPR, thereby limiting its overall effectiveness.^3^, ^4^, ^5^, ^6^, ^7^ By contrast, recent advances combining active chest compression-decompression CPR, an impedance threshold device (ITD), and a controlled, gradual head and thorax elevation, also known as automated head-up CPR (AHUP-CPR), have demonstrated improved hemodynamics parameters, brain blood flow, and survival in animal models compared with C-CPR.^8^, ^9^, ^10^, ^11^, ^12^, ^13^ Use of AHUP-CPR in patients is associated with a significant time-dependent survival benefit when compared with C-CPR, regardless of the presenting heart rhythm.^14^, ^15^

Resuscitative endovascular balloon occlusion of the aorta (REBOA) has emerged as another promising adjunctive intervention also capable of increasing blood flow to the heart and brain during CPR.^16^, ^17^ In animal models of cardiac arrest, REBOA combined with C-CPR has been reported to enhance coronary and cerebral perfusion pressures and improve the likelihood of return of spontaneous circulation (ROSC).^18^ However, deflation of the REBOA balloon after ROSC may be associated with sudden decrease in hemodynamics and re-arrest. This may have been the case in some pilot clinical studies where the incidence of ROSC was higher with REBOA, but overall survival was still poor.^18^, ^19^, ^20^ One experimental study utilizing C-CPR has recently shown that an automated, and controlled deflation of the REBOA balloon after C-CPR and ROSC improved and stabilized blood pressures, preventing re-arrest.^21^ However, an optimal strategy to safely deflate a REBOA balloon during post-ROSC care is not known. This may be particularly important after AHUP-CPR, if the head and thorax remain elevated after ROSC in an effort to reduce intracranial pressures and optimize post-ROSC hemodynamics.^22^

To address this knowledge gap, the primary aim of this study was to compare the immediate hemodynamic consequences of complete (100%) versus partial (50%) REBOA balloon deflation following prolonged AHUP-CPR and ROSC in a porcine model of cardiac arrest. We hypothesized that partial deflation would better preserve overall hemodynamics, including cerebral, coronary, and systemic perfusion pressures immediately after ROSC.

## Methods

Study protocols for animals used in this study were approved by the Institutional Animal Care Committee of Hennepin Healthcare Research Institute (IACUC Protocol Numbers 20-10 and 21-08) and were compliant with the National Research Council’s 2011 Guidelines for the Care and Use of Laboratory Animals. This study was conducted and reported in accordance with the ARRIVE guidelines 2.0 (the full checklist is provided in Appendix C).^23^

### Study design

This study focused on the REBOA deflation phase post-ROSC using individual animal data from two prior investigations with similar CPR intervention protocols: one focused on different ventilation strategies and the other on different compression waveform strategies. A REBOA catheter was used in both prior studies as an adjunct to AHUP-CPR. Inclusion criteria for the current post-ROSC REBOA deflation study included all pigs from those two prior protocols that achieved ROSC and underwent REBOA deflation. Protocol details are described below.

### Study objectives and endpoints

The primary objective of this study was to compare the hemodynamic consequences of complete (100%) versus partial (50%) REBOA balloon deflation, assessed one minute before and one minute after deflation, following prolonged AHUP-CPR and ROSC in a porcine model of cardiac arrest.

The primary endpoint was the change in mean arterial pressure (MAP) before and after deflation (comparing partial versus complete deflation). Secondary endpoints included changes in cerebral perfusion pressure (CerPP), coronary perfusion pressure (CorPP), systolic and diastolic arterial (SAP and DAP), intracranial (ICP), and right atrial (RAP) pressures, and end-tidal CO_2_ (EtCO_2_). Additionally, secondary endpoints included baseline hemodynamic parameters 5 min before ventricular fibrillation (VF) induction, as well as hemodynamic parameters 1 min before and 1 min after REBOA inflation during CPR.

### Animal preparation and CPR protocol

The details of animal surgical preparation and CPR performance have been previously described.^9^, ^12^, ^13^ Male and female Yorkshire-Duroc farm juvenile pigs, provided by a local licensed breeding facility, weighing approximately 40 ± 2 kg, were house in the animal care facility adjacent to the laboratory. Following an acclimatization period of 2–5 days, they were anesthetized and ventilated. Anesthesia was induced with intramuscular ketamine (20–30 mg/kg) and maintained with inhaled isoflurane (1–2%) after intubation with a 7.5 mm cuffed endotracheal tube (ETT). Ventilation was set to a tidal volume of 8 ml/kg (Narkomed, North American Dräger). Proximal airway pressure was monitored using a differential pressure transducer (TSD160C, BioPac Systems, Inc.) mounted on the ETT. A pressure transducer (Mikro-Tip transducer, Millar Inc, Houston, TX, USA) was inserted through a burr hole created over the parietal lobe to measure ICP. After venous and arterial femoral vascular access was established on both sides (left and right), micromanometer-tipped catheters (Mikro-Tip Transducer, Millar Instruments) measured pressures in the descending thoracic aorta (AoP) and right atrium (RAP). Catheter placements were confirmed by fluoroscopy. A 1000 cc bolus of normal saline solution was administered during the preparatory phase to maintain the RAP between 7 and 10 mmHg. Body temperature was maintained between 36.5 and 38.5 °C. Continuous intravascular monitoring and recording (BioPac; BioPac Systems Inc.) included aortic pressures, venous pressures, surface electrocardiograms, and EtCO_2_. Heparin for venous thrombus prophylaxis was administered intravenously, starting with a 5000-unit bolus, followed by 1000 units hourly.

VF was induced by delivering direct intracardiac current via a temporary pacing wire under fluoroscopy. After 10 min of untreated VF, C-CPR was performed for 2 min, AHUP-CPR was then performed for a period of 2 min with active chest decompression and ITD (ResQPOD-16 TM ZOLL Medical) with the head and thorax minimally elevated (relative to horizontal plane, thorax was elevated 8 cm and the head was raised 10 cm), followed by 2 min of gradual head and thorax elevation to the maximum head and thorax elevation position. Full elevation, with the head at 22 cm and thorax at 10 cm, was achieved after 6 min total of CPR.^9^ Once the pigs were in the full elevation position, AHUP-CPR was continued for 40 or 50 min, depending on the protocol. Throughout CPR, animals were ventilated with a customized automated resuscitator bag compressor that delivered a breath over 1 s with a tidal volume of 8 ml/kg and supplemental oxygen, as needed, to maintain SpO_2_ >92%.^24^ Breaths were delivered with a 10:1 compression: ventilation rate. A 100 mg bolus of intravenous succinylcholine (2.5 mg/kg) was administered at the start and, if needed, during CPR, to intentionally inhibit spontaneous gasping. A REBOA catheter (a semi-compliant 7-Fr radio-opaque REBOA catheter with a balloon length of approximate 1.5 in (3.8 cm) requiring ∼10 cc of saline to fully inflate and occlude the aorta, from Certus Critical Care Inc.®, Salt Lake City, USA), positioned in Zone 1 at the level of the diaphragm in the descending aorta, and was inflated after a median time of 36 min. REBOA Zone 1 positioning was confirmed via fluoroscopy prior to CPR in all pigs. Complete balloon occlusion of the aorta upon REBOA inflation was verified in some but not all pigs by the profound decrease in aortic pressure distal to the balloon. A representative tracing demonstrating the reduction in aortic blood pressure with balloon inflation is shown in Appendix B. Depending on the protocol, 4 min (ventilation study) or 14 min (waveform comparison study) after balloon inflation epinephrine (0.5 mg) and amiodarone (25 mg) were given, and the pigs were defibrillated with a 360j shock (LifePack15, Stryker®, USA).

### Post-ROSC protocol

After ROSC the pig’s head and thorax remained in the elevated position, with the head and heart 22 and 10 cm above the horizontal plane, as previously described.^9^, ^12^, ^13^ The REBOA was then randomly deflated in two ways: a full and complete deflation over 10 s in one group and a 50% deflation assessed by volume removed from the balloon (partial deflation) over 10 s, in the other group. Deflation was performed using a syringe and the amount of deflation was based on the volume of saline solution that was used for inflation. The key hemodynamics compared in this report was those measured one minute before and one minute after REBOA deflation.

### Statistical analysis

Continuous variables were expressed as mean ± standard deviation (SD). The primary analysis focused on the absolute change in hemodynamic parameters before and after REBOA deflation.

The Mann-Whitney *U* test was used to compare the 50% deflation group and the 100% deflation group. The results are presented as mean differences accompanied by their 95% confidence intervals (CI). A two-tailed *p*-value < 0.05 was considered statistically significant for the primary outcome (MAP). Secondary outcomes (SAP, DAP, CerPP, CorPP, ICP, RAP, and EtCO_2_) comparing the two groups were analyzed using the same methodology. Parameters measured during CPR were analyzed using a Wilcoxon Signed-Rank Test (for non-parametric paired data). In accordance with the exploratory nature of this post-hoc analysis, no correction for multiple comparisons was applied. All statistical analyses were performed using Jamovi software (Version 2.3.21.0).

## Results

### Study population

A total of 20 pigs were treated with REBOA after prolonged AHUP-CPR and 13 achieved ROSC. There were 5 pigs (38%) in the partial deflation group and 8 (62%) in the complete deflation group (Appendix A). The population included 6 males (46%) and 7 females (54%). No significant differences were observed in the mean CPR duration between the partial and complete deflation groups (43.2 ± 11.8 min in the 50% group vs. 45.3 ± 8.4 min in the 100% deflation group; *p* = 0.6). The mean dose of epinephrine administered during CPR was similar between groups (0.3 ± 0.4 mg in the partial deflation group and 0.4 ± 0.3 mg in the complete deflation group, *p* = 0.47). After ROSC, the mean time to start balloon deflation (partial or complete) was 9.3 ± 7.7 min in the partial deflation group compared to 3.9 ± 1.4 min in the complete deflation group (*p* = 0.37).

### Baseline and CPR measurements

In pigs that achieved ROSC, REBOA inflation after ∼35 min of CPR resulted in a statistically significant increase in MAP, from 53.7 ± 12.0 mmHg to 78.2 ± 17.9 mmHg (mean difference 24.5 mmHg; 95% CI 10.4–34.4; *p* < 0.001) as well as systolic arterial pressure (SAP), from 82.1 ± 14.3 mmHg to 119.7 ± 29.2 mmHg (mean difference 37.6 mmHg; 95% CI 13.7–62.3; *p* < 0.001). CerPP also improved significantly, increasing from 37.7 ± 10.5 mmHg to 55.8 ± 16.4 mmHg (mean difference 18.1 mmHg; 95% CI 4.95–27.5; *p* = 0.002). Conversely, while DAP and coronary perfusion pressure (CoPP) also increased by 11.4 mmHg and 11.6 mmHg respectively, these changes did not reach statistical significance (*p* = 0.09 and *p* = 0.30). No significant changes were observed in RAP, ICP, or EtCO_2_ following REBOA inflation (Table 2). These data are also synthesized in Fig. 3.

### Post-ROSC hemodynamic measurements

**Fig. 1:**
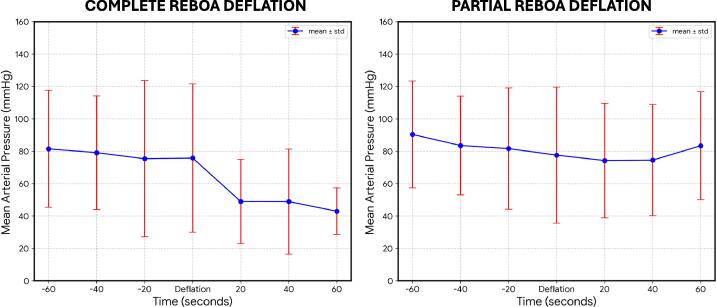
**Continuous plot of mean arterial pressure during complete (100%) versus partial (50%) REBOA deflation following return of spontaneous circulation (ROSC)**. *The mean arterial pressure (mean ± std) were sampled every 20 s one minute before and one minute after REBOA deflation.*

**Fig. 2:**
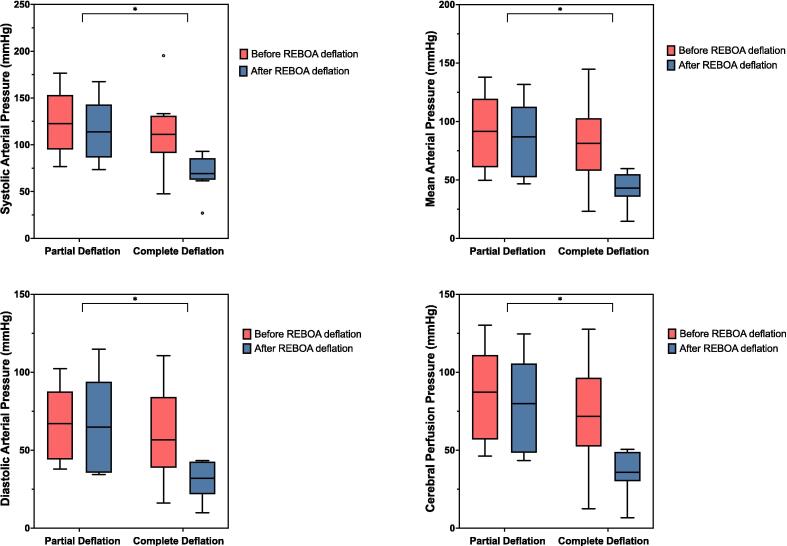
**Box plots representing key hemodynamic parameters 1 min before (pink) and 1 min after (blue) REBOA deflation. Data are shown for both the complete (100%) and partial (50%) deflation groups**. Statistical analyses were performed using a Mann-Whitney U test to compare the differences (before vs. after) between the two groups (100% vs. 50% deflation). An asterisk (*) indicates a significant difference (p < 0.05). The data show point-in-time measurements from 1 min prior to and 1 min following REBOA deflation. (For interpretation of the references to color in this figure legend, the reader is referred to the web version of this article.)

Hemodynamic results are summarized in Table 1, and box plots illustrating the primary hemodynamic endpoint parameters are presented in Fig. 2. Continuous mean arterial pressure recordings are shown in Fig. 1.

**Table 1:** Comparison of hemodynamic parameters before and after REBOA deflation after return to spontaneous circulation.

**Hemodynamic parameter (mmHg)**	**Deflation group**	**BEFORE** **REBOA deflation** **(mean ± std)**	**AFTER** **REBOA deflation** **(mean ± std)**	**Mean difference with 95% CI**	***p***
Mean arterial pressure (MAP)	Partial	90.4 ± 33.0	83.4 ± 33.3	7.0 (1.8–12.3)	**0.006**
	Complete	81.5 ± 36.1	43.0 ± 14.4	38.5 (17.0–60.0)	

Systolic arterial pressure (SAP)	Partial	123.9 ± 35.9	114.5 ± 34.4	9.4 (4.4–14.4)	**0.02**
	Complete	113.7 ± 42.0	69.0 ± 20.2	44.7 (18.2–71.2)	

Diastolic arterial pressure (DAP)	Partial	66.1 ± 24.5	64.8 ± 32.6	1.3 (−10.3 to 12.8)	**0.006**
	Complete	61.0 ± 29.7	31.3 ± 11.9	29.7 (11.9–47.4)	

Cerebral perfusion pressure (CerPP)	Partial	84.6 ± 31.2	77.6 ± 31.8	7.1 (1.7–12.5)	**0.01**
	Complete	72.3 ± 34.4	35.9 ± 14.6	36.4 (15.9–56.8)	

Coronary perfusion pressure (CoPP)	Partial	83.0 ± 32.7	76.1 ± 33.0	6.9 (0.9–11.2)	**0.006**
	Complete	74.1 ± 37.3	36.1 ± 15.8	38.0 (18.7–60.9)	

Right atrial pressure (RAP)	Partial	7.5 ± 2.1	7.3 ± 2.2	0.2 (−0.1 to 0.4)	0.17
	Complete	7.4 ± 2.6	6.9 ± 2.8	0.6 (0.07–1.07)	

Intracranial pressure (ICP)	Partial	6.0 ± 5.1	5.8 ± 5.1	0.2 (−0.2 to 0.6)	**0.04**
	Complete	8.9 ± 4.2	7.1 ± 2.9	1.8 (0.14–3.52)	

End-tidal CO_2_ (EtCO_2_)	Partial	34.8 ± 8.4	33.3 ± 10.1	1.5 (−3.6 to 6.8)	0.94
	Complete	34.6 ± 12.4	34.4 ± 16.1	0.2 (−3.7 to 4.0)	

*All statistical analyses were performed using a Mann-Whitney U test*.

**Table 2:** Hemodynamic parameters prior to CPR and during CPR before and after REBOA inflation.

**Hemodynamic parameter (mmHg)**	**Baseline before ventricular fibrillation** **(mean ± std)**	**BEFORE** **REBOA inflation** **(mean ± std)**	**AFTER** **REBOA inflation** **(mean ± std)**	**Mean difference with 95% CI between after and before REBOA inflation**	***p***
Mean arterial pressure (MAP)	81.1 ± 11.9	53.7 ± 12.0	78.2 ± 17.9	24.5 (10.4–34.4)	**<0.001**
Systolic arterial pressure (SAP)	103.6 ± 14.1	82.1 ± 14.3	119.7 ± 29.23	37.6 (13.7–62.3)	**<0.001**
Diastolic arterial pressure (DAP)	69.8 ± 11.8	25.3 ± 14.4	36.7 ± 21.1	11.4 (−3.81 to 27.2)	0.09
Cerebral perfusion pressure (CerPP)	65.7 ± 11.7	37.7 ± 10.5	55.8 ± 16.4	18.1 (4.95–27.5)	**0.002**
Coronary perfusion pressure (CoPP)	77.3 ± 14.2	20.5 ± 17.7	32.1 ± 16.5	11.6 (−4.69 to 26.3)	0.30
Right atrial pressure (RAP)	8.7 ± 4.1	17.4 ± 8.7	22.3 ± 8.3	4.9 (−2.64 to 12.9)	0.17
Intracranial pressure (ICP)	20.3 ± 3.8	11.7 ± 8.0	11.6 ± 6.3	−0.1 (−5.47 to 6.22)	0.83
End-tidal CO_2_ (EtCO_2_)	43.2 ± 3.8	29.6 ± 9.0	28.6 ± 7.6	−1.0 (−7.89 to 5.64)	0.85

*Measurements were made 5 min before initiation of ventricular fibrillation, 1 min before REBOA inflation and 1 min after REBOA inflation*.

*All statistical analysis was performed using a Wilcoxon Signed-Rank Test*.

**Fig. 3:**
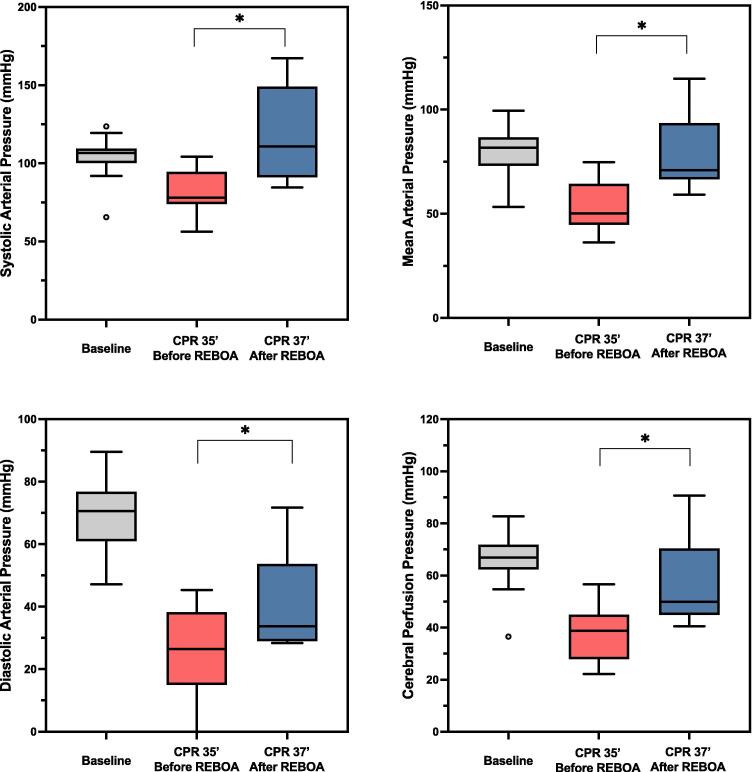
**Box plots representing key hemodynamic parameters at three time points: before CPR (baseline, gray); during CPR, 1 min before REBOA inflation (pink); and during CPR, 1 min after REBOA inflation (blue)**. Statistical analyses were performed using a Wilcoxon Signed-Rank Test to compare differences before versus after REBOA inflation. An asterisk (*) indicates a significant difference (p < 0.05). (For interpretation of the references to color in this figure legend, the reader is referred to the web version of this article.)

Regarding the primary outcome, mean arterial pressure (MAP) in the complete deflation group decreased from 81.5 ± 36.1 mmHg before deflation to 43.0 ± 14.4 mmHg after deflation. By comparison, in the partial deflation group MAP remained more stable, decreasing from 90.4 ± 33.0 mmHg before deflation to 83.4 ± 33.3 mmHg after. This represented a significantly greater mean decrease in the complete deflation group (38.5 mmHg; 95% CI: 17.0–60.0) compared to the partial deflation group (7.0 mmHg; 95% CI: 1.8–12.3; *p* = 0.006).

Results were similar for secondary outcomes (Table 1). The mean decrease in systolic arterial pressure (SAP) was significantly lower in the partial deflation group (9.4 mmHg (95% CI: 4.4–14.4)) than in the complete deflation group (44.7 mmHg (95% CI: 18.2–71.2); *p* = 0.02). Significant differences were also observed for diastolic arterial pressure (DAP) (1.3 mmHg (95%CI: −10.3 to 12.8) in the partial deflation group vs. 29.7 mmHg (95%CI: 11.9–47.4) in the complete deflation group; *p* = 0.006) and cerebral perfusion pressure (CerPP) (7.1 mmHg (95%CI: 1.7–12.5) in the partial deflation group vs. 36.3 mmHg (95%CI: 15.9–56.8) in the complete deflation group; *p* = 0.01). The mean difference in intracranial pressure (ICP) was 0.2 mmHg (95%CI: −0.2 to 0.6) in the partial deflation group compared to 1.8 mmHg (95%CI: 0.14–3.52) in the complete deflation group (*p* = 0.04). No significant differences were observed between groups for RAP or EtCO_2_.

## Discussion

The application of REBOA to improve hemodynamics during CPR has recently gained significant attention. The present study was focused on the potential challenges associated with deflation of REBOA after ROSC. The results from this study demonstrate that the strategy utilized to deflate the REBOA balloon following AHUP-CPR and ROSC significantly impacts hemodynamic stability. Immediate and complete deflation resulted in an immediate, significant, and life-threatening deterioration in mean, systolic, and diastolic arterial pressures, as well as cerebral and coronary perfusion pressures. In contrast, a rapid but partial deflation was associated with a significantly more stable hemodynamic profile, preserving both systemic and cerebral perfusion. These findings highlight the critical risk of abrupt cardiovascular collapse and potential for re-arrest when the balloon is fully removed too rapidly.

Physiologically, these new findings suggest that an initial 50% reduction in balloon volume is hemodynamically well-tolerated. This threshold appears to be a safe initial step in the transition from full aortic occlusion to un-occluded circulation. The stability observed at 50% deflation suggests that the most critical hemodynamic inflection point occurs during the latter stages of deflation (from 50% to 0%).

The current findings may be helpful in explaining some of the recent results from other groups reporting REBOA use. For instance, Jang et al. reported ROSC in 6 of 15 patients, Daley et al. reported 4 of 5 patients, and Gamberini et al. observed sustained ROSC in 5 of 18 patients with traumatic and non-traumatic cardiac arrest.^18^, ^19^, ^20^ Recently, the REBOARREST trial, including 179 patients, of which 88 patients in the REBOA group, found a ROSC rate of 28% and a 30-day survival rate of 7%.^25^ Despite improved rates of ROSC, survival to discharge remains extremely low. This high mortality is frequently linked to re-arrest upon REBOA deflation. One potential reason for this could be a rapid decrease in MAP, as observed in the current pig study. Neither the heart nor the vasculature appears to be able to sufficiently compensate for a sudden decrease in afterload with 100% REBOA deflation shortly after ROSC. The challenges of post-ROSC hypotension were recently studied in an experimental pig model of study of cardiac arrest and conventional CPR with REBOA.^21^ In that study, automated, controlled deflation was used to try to mitigate the risks of cardiovascular collapse. The authors compared two strategies, one with automated, dynamic adjustments to maintain a DBP of 60 mmHg versus a second wherein the balloon was deflated steadily over 5 min (control). No re-arrest occurred in the group with automated, dynamic adjustments, whereas 60% (3/5) of the control group re-arrested. Another group recently proposed using transesophageal echocardiography during the post-ROSC phase to assess cardiac afterload and guide deflation.^26^ While such future opportunities are anticipated to provide better strategies,^27^, ^28^, ^29^ current post-ROSC protocols are often poorly described and lack standardization, leaving decisions to the discretion of the attending physician. The present study is the first to describe the challenges of REBOA deflation after AHUP-CPR, and reinforces the urgent need for a dedicated, standardized protocol to prevent the hemodynamic deterioration that can lead to early re-arrest. Maintaining partial occlusion while titrating vasopressors or fluids before a progressive complete deflation is one strategy, as described herein, that merits further evaluation.

In addition to focusing on the challenges of deflation during AHUP-CPR, the current results also assessed some of the physiologic effects of REBOA inflation during AHUP-CPR. The REBOA balloon was inflated in the current study on average 36 min after the start of AHUP-CPR (46 min after VF induction), to determine if it could be of added value in the prolonged arrest setting. REBOA inflation significantly increased SAP, MAP, and CerPP and in 13/20 pigs ROSC was achieved. In the pigs that achieved ROSC coronary perfusion pressure also increased with REBOA inflation from 20.5 ± 17.7 mmHg to 32.1 ± 16.5 mmHg, (*p* = 0.30), but this increase was not statistically significant. Further research is ongoing to investigate the potential contribution of REBOA to AHUP-CPR.

### Limitations

Limitations are acknowledged. First, this was a post-hoc analysis of data from two previous studies, not a prospective trial designed specifically to compare post-ROSC deflation strategies. Due to the exploratory nature of this study, no correction for multiple comparisons was applied. Consequently, the risk of Type I errors is increased, and the results should be interpreted accordingly. Second, the sample size was relatively small (*n* = 13), which may limit the generalizability of the findings. The new observations nonetheless provide strong evidence that post-ROSC REBOA deflation should be considered as a potential risk that needs to be managed if REBOA is going to be widely used in the treatment of cardiac arrest, whether during conventional or AHUP-CPR. Third, while previous research indicates that head and thorax elevation during the post-ROSC phase can decrease intracranial pressure and increase cerebral blood flow,^22^ such positioning may also influence afterload and exacerbate the hemodynamic effects of REBOA deflation. Further studies are required to compare REBOA efficacy in the head-up versus flat position.

Fourth, the animals underwent a very prolonged CPR duration (mean of 45 min). While this reflects worst-case clinical scenarios where adjunctive therapies like AHUP-CPR and REBOA are often deployed, the hemodynamic response to REBOA deflation during post-ROSC might be more favorable in scenarios with shorter CPR durations.^30^ Additionally, it was not possible to assess longer-survival outcomes after deflation, as this outcome was not part of the initial protocols.

Fifth, there seems to be potential key physiological factors that can determine if hypotension will occur with REBOA balloon deflation including: speed of deflation, time to deflation, MAP before and during deflation process, concurrent use of vasopressor, the severity of the injury (duration of no-flow and low-flow time during CPR) and method of CPR. Moreover, the reduction of balloon volume and hemodynamic effect are not necessarily linear. The balloon deflation seems to be linked to the balloon compliance, vessel diameter, positioning and vascular tone. A better assessment of these parameters should be done in future studies. The time from ROSC to the initiation of REBOA deflation also differed by five minutes between groups, potentially favoring the partial deflation group. While this disparity could influence the study findings, a five-minute difference is likely negligible within the broader context of post-ROSC management. As we have continued to study this issue, there continues to be a threshold value of balloon deflation, around 50%, where there remains a high risk for a marked decrease in MAP, even in the presence of vasopressors, regardless of when the deflation is performed within 15 min post-ROSC. MAP in the post-ROSC period, even while the REBOA is inflated, can be a clinically challenging time due to hemodynamic instability. The optimal deflation rate and time and how to guide this process should be more rigorously documented and investigated in future research. Finally, we were unable to measure lactate levels and other markers of tissue metabolism, or arterial pressures distal to the REBOA balloon during deflation in all, but just some of the pigs. Such metabolic and physiologic parameters, including comparisons of different targets and management strategies for REBOA balloon deflation during post-ROSC, should be evaluated in future studies to provide a more comprehensive understanding of the deflation phase.

## Conclusion

In this post-hoc analysis of experimental porcine studies, following prolonged AHUP-CPR, a 50% partial deflation of the REBOA balloon provided superior hemodynamic stability compared to a 100% complete deflation. These findings emphasize the importance of management of the REBOA balloon deflation after ROSC. Ultimately a controlled, progressive, and perhaps feed-back loop-controlled REBOA deflation strategy may optimally preserve hemodynamics stability and to improve the chances of sustained survival after cardiac arrest.

## CRediT authorship contribution statement

**N. Segond:** Writing – original draft, Visualization, Validation, Methodology, Investigation, Formal analysis, Conceptualization. **J. Moore:** Writing – review & editing, Validation, Supervision, Methodology, Investigation, Conceptualization. **P. Pourzand:** Writing – review & editing, Investigation, Conceptualization. **B. Salverda:** Writing – review & editing, Investigation, Conceptualization. **M. Suresh:** Writing – review & editing, Investigation. **K. Bachista:** Writing – review & editing. **A. Johnson:** Writing – review & editing, Validation, Resources. **S.T. Youngquist:** Writing – review & editing, Validation, Resources. **G. Debaty:** Writing – review & editing, Validation, Supervision, Methodology. **K. Lurie:** Writing – review & editing, Writing – original draft, Validation, Supervision, Resources, Methodology, Conceptualization.

## Funding

Nicolas Segond gratefully acknowledges financial support for this publication by the Fulbright Program, which is sponsored by the U.S. Department of State, the Franco-American Commission – Fulbright France and Grenoble-Alpes University. Its contents are solely the responsibility of the author and do not necessarily represent the official views of the Fulbright Program, the Government of the United States, or the Franco-American Commission.

## Declaration of competing interest

The authors declare the following financial interests/personal relationships which may be considered as potential competing interests: Dr. Keith Lurie is the Chief Medical Officer of AdvancedCPR Solutions, the company that makes a human patient positioning system for Head Up CPR. While the device used for this porcine research is not one that can be used in humans, the relationship with AdvancedCPR Solutions is a potential conflict of interest. Dr. Lurie did not receive any funding from Zoll or Stryker for their prior work developing the ACD + ITD CPR adjuncts.

Dr. Austin Johnson is the Chief Medical Officer of Certus Critical Care, the company that made the REBOA catheters used in this study. Dr. Johnson was not directly involved with performing any of the actual experiments or performing any of the data analysis, but was involved in helping to conceptualize the study and interpret the results.

Pr. Guillaume Debaty is a member of the International Liaison Committee on Resuscitation (ILCOR) in the basic-life support group.

No other co-authors have any financial or competing conflicts of interest.
