# Supplementary material for: Physiological changes after deflation of Resuscitative Endovascular Balloon Occlusion of the Aorta following Automated Head-up Position Cardiopulmonary Resuscitation: a post-hoc analysis
Source: Resusc Plus. 2026 May 27;30:101370. doi: 10.1016/j.resplu.2026.101370 (PMC13264273; doi:10.1016/j.resplu.2026.101370)

**APPENDIX**

**Appendix A – Study flow-chart**

**Appendix B – Example of REBOA balloon inflation validation**

**(blue curve: aortic pressure in mmHg measured with a Millar catheter, red curve: distal aortic pressure in mmHg measured using a hydrostatic pressure sensor).**

**Appendix C – ARRIVE 2.0 guidelines author checklist**


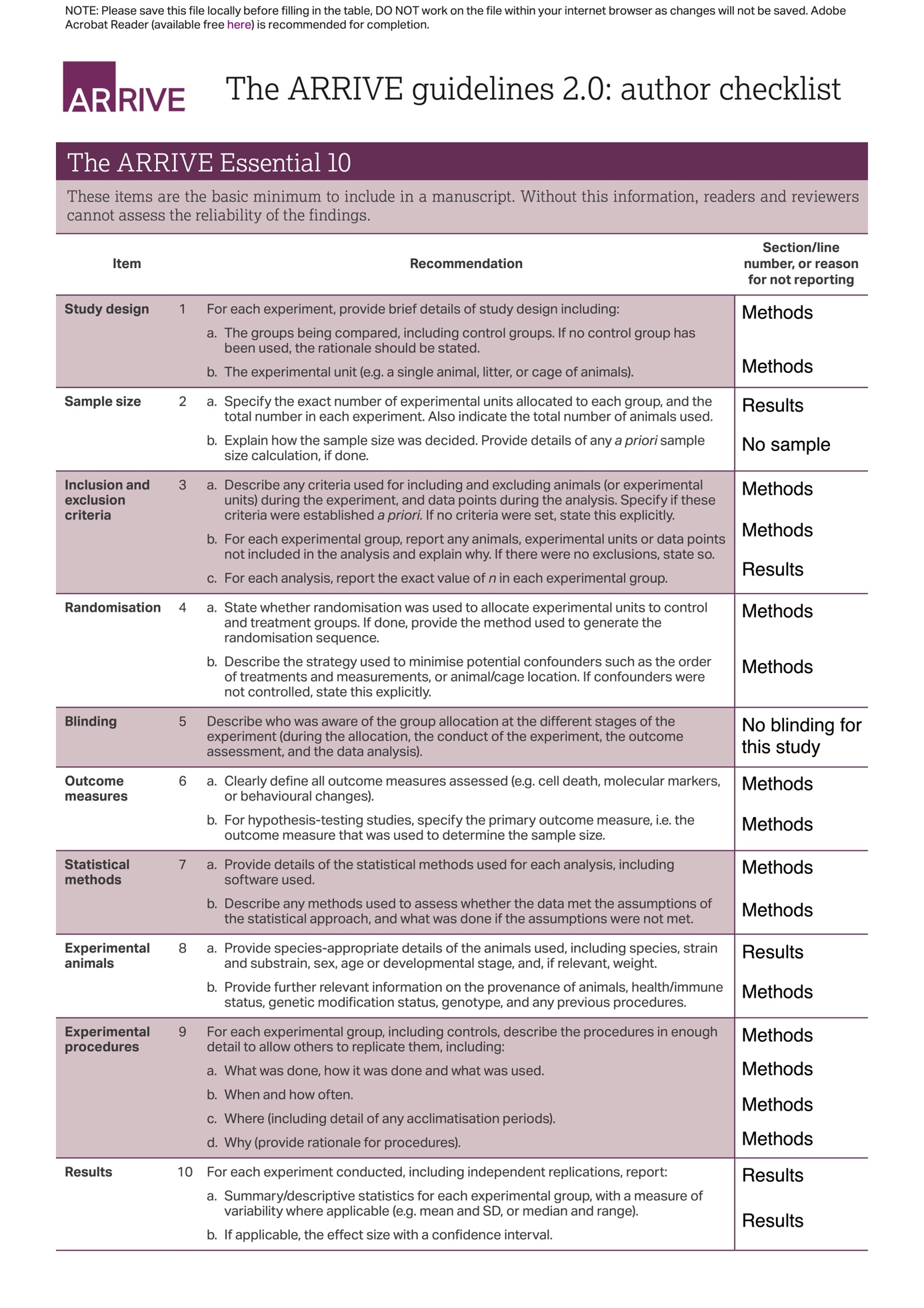


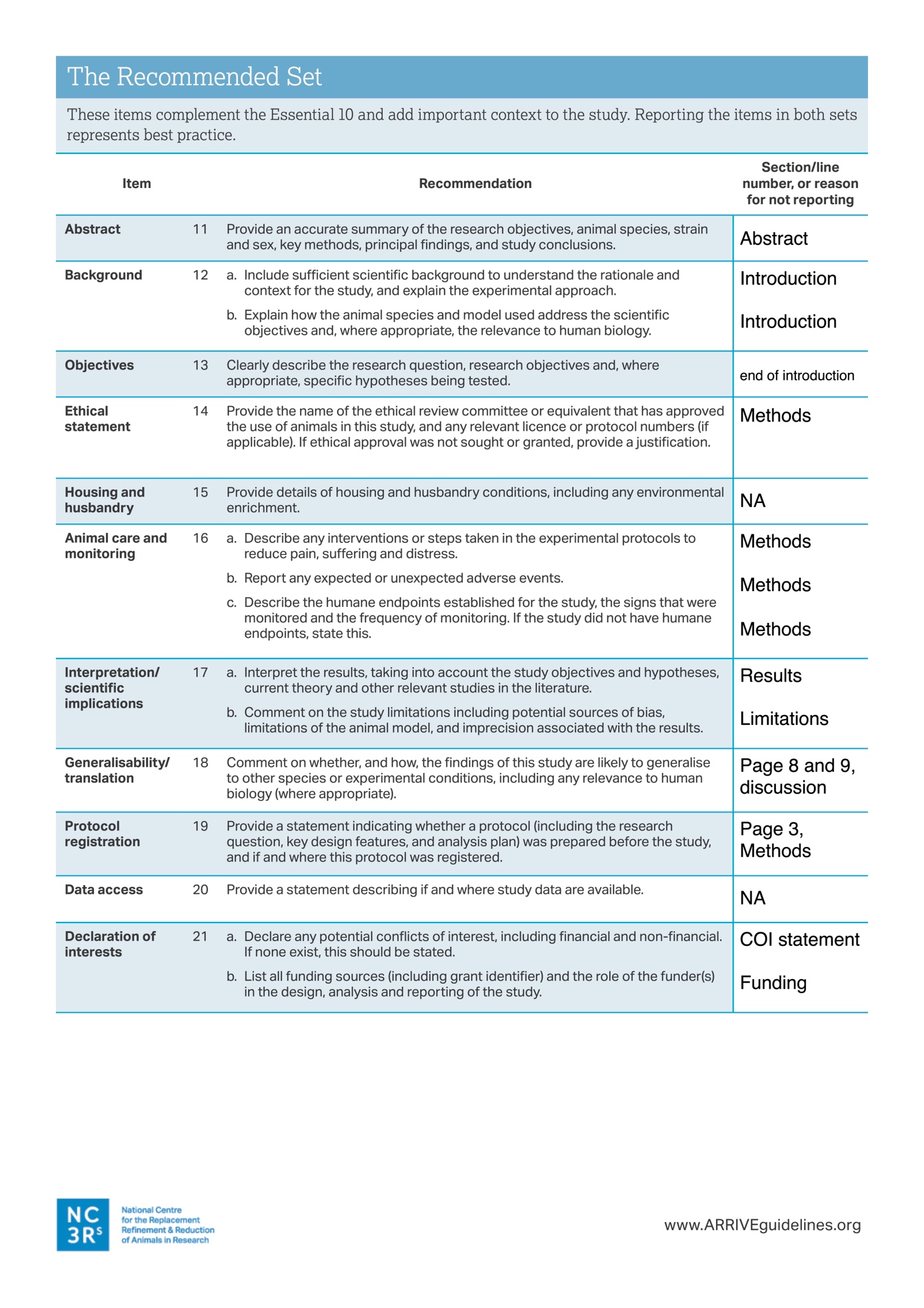

Supplement: Supplementary Data 1 [file mmc1.docx]
